# Molecular Tumor Boards in Malignant Melanoma: Uncovering Challenges and Opportunities in a Bicenter Retrospective Analysis in Germany

**DOI:** 10.1002/ijc.70554

**Published:** 2026-05-15

**Authors:** Glenn Geidel, Theresa Lowinus, Patrick Metzger, Rebecca Czurda, Vincent Schipperges, Ulrike Paul, Alessandra Rünger, Julian Kött, Isabel Heidrich, Saskia Lehr, Julia Kühn, Heiko Becker, Cornelius Miething, Martin Werner, Silke Laßmann, Justus Duyster, Tilman Brummer, Ronald Simon, Winfried Alsdorf, Maximilian Christopeit, Carsten Bokemeyer, Kilian Eyerich, Stefan W. Schneider, David Rafei, Melanie Börries, Frank Meiss, Christoffer Gebhardt

**Affiliations:** ^1^ Department of Dermatology and Venereology University Medical Center Hamburg‐Eppendorf Hamburg Germany; ^2^ Fleur Hiege Center for Skin Cancer Research University Medical Center Hamburg‐Eppendorf Hamburg Germany; ^3^ Department of Medicine I, Medical Center—University of Freiburg, Faculty of Medicine University of Freiburg Freiburg Germany; ^4^ Institute of Medical Bioinformatics and Systems Medicine, Medical Center—University of Freiburg, Faculty of Medicine University of Freiburg Freiburg Germany; ^5^ Institute of Pathology University Medical Center Hamburg‐Eppendorf Hamburg Germany; ^6^ Institute of Tumor Biology University Medical Center Hamburg‐Eppendorf Hamburg Germany; ^7^ Department of Dermatology and Venereology, Medical Center—University of Freiburg, Faculty of Medicine University of Freiburg Freiburg Germany; ^8^ German Cancer Consortium (DKTK), Partner Site Freiburg; and German Cancer Research Center (DKFZ) Heidelberg Germany; ^9^ Comprehensive Cancer Center Freiburg, Medical Center—University of Freiburg, Faculty of Medicine University of Freiburg Freiburg Germany; ^10^ Institute for Surgical Pathology, Medical Center—University of Freiburg, Faculty of Medicine University of Freiburg Freiburg Germany; ^11^ Institute of Molecular Medicine and Cell Research Centre of Biochemistry and Molecular Cell Research (ZMBZ) University of Freiburg Freiburg Germany; ^12^ University Cancer Center Hamburg University Medical Center Hamburg‐Eppendorf Hamburg Germany; ^13^ Department of Oncology, Hematology and Bone Marrow Transplantation With Section Pneumonology University Medical Center Hamburg‐Eppendorf Hamburg Germany; ^14^ Center for Personalized Medicine—Oncology University Medical Center Hamburg‐Eppendorf Hamburg Germany

**Keywords:** clinical decision‐making, melanoma, molecular tumor board, personalized medicine, precision oncology

## Abstract

Despite therapeutic advancements, approximately 50% of advanced melanoma patients succumb to metastatic disease. Molecular tumor boards (MTB) aim to identify targetable molecular alterations to guide individualized treatment strategies. Yet, real‐world data on patient selection, referral timing, recommendation rates, implementation, and clinical impact remain limited. In this exploratory retrospective bicenter analysis, we evaluated 80 patients with advanced melanoma who presented at institutional MTBs of two comprehensive cancer centers. Clinical and molecular tumor data were analyzed using bioinformatic tools to characterize mutation profiles, treatment recommendations, and their real‐world implementation. Most patients (88.3%) had stage IV melanoma at the time of presentation and had received a median of three prior systemic treatment lines. Actionable treatment recommendations were formulated in 77.9% of eligible cases, yet only 33.7% of recommendations were implemented. Non‐implementation was most commonly attributable to early patient death or regulatory barriers. Importantly, when recommended therapies were applied, patients experienced significantly improved progression‐free survival (7.85 vs. 4.34 months; PFS ratio 1.8) and overall survival (10.64 vs. 5.06 months) compared with patients in whom recommended treatments were not implemented. Among patients with implemented MTB recommendations (*n* = 26), the median intra‐patient PFS ratio was 1.68, and 14 of 26 patients (53.8%) achieved a PFS ratio ≥ 1.3. These findings indicate that MTBs frequently generate clinically actionable recommendations for metastatic melanoma, but late‐stage referral substantially limits their real‐world implementation. When applied, molecularly guided treatment strategies may confer meaningful clinical benefit, underscoring the importance of earlier integration of MTBs into melanoma care pathways.

## Abstract (highlights)

What's new?

Molecular tumor boards (MTB), interdisciplinary teams that use tumor genomic data to guide personalized treatment decisions, have emerged as a promising strategy in melanoma care, although their real‐world clinical impact remains uncertain. This retrospective study evaluated advanced melanoma patients to assess molecularly guided treatment recommendations, implementation, and outcomes. Actionable recommendations were generated for most eligible patients, but only one‐third of recommendations were implemented, largely due to late referral or regulatory barriers. Patients who received recommended therapies experienced significantly longer survival outcomes. The findings highlight the value of MTBs and underscore the need for incorporating them into early melanoma care.

## Graphical abstract

Molecular tumor boards (MTB), interdisciplinary teams that use tumor genomic data to guide personalized treatment decisions, have emerged as a promising strategy in melanoma care, although their real‐world clinical impact remains uncertain. This retrospective study evaluated advanced melanoma patients to assess molecularly guided treatment recommendations, implementation, and outcomes. Actionable recommendations were generated for most eligible patients, but only one‐third of recommendations were implemented, largely due to late referral or regulatory barriers. Patients who received recommended therapies experienced significantly longer survival outcomes. The findings highlight the value of MTBs and underscore the need for incorporating them into early melanoma care. Created in BioRende. Geidel, G (2026) https://BioRender.com/sxbfd0j.

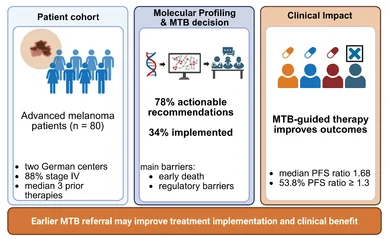

**Abbreviations**
ECOG: Eastern cooperative oncology group
ICI: Immune checkpoint inhibitor
MAF: Mutation annotation format
MTB: Molecular tumor board
NGS: Next generation sequencing
OS: Overall survival
PFS: Progression‐free survival
SNP: Single nucleotide polymorphism
SNV: Single nucleotide variant
TCGA: The cancer genome atlas
TMB: Tumor mutational burden

## 1 Introduction

Malignant melanoma is a highly aggressive skin cancer with early metastasis and poor prognosis in advanced stages. Despite systemic therapy advancements, including immune checkpoint inhibitors (ICI) and targeted treatments, approximately 50% of patients with metastatic melanoma succumb to the disease [1]. While BRAF and MEK inhibitors provide effective options for patients with actionable BRAF mutations, resistance formation is common, necessitating novel treatment strategies [2, 3]. Additionally, therapeutic options remain limited for those with rare, uncharacterized, or unknown molecular alterations.

Recruitment into clinical trials exploring novel targeted or immunotherapeutic approaches is a potential avenue for these patients. However, many trials are restrictive, with patients often ineligible due to prior treatments, treatment‐related events, comorbidities, or specific biomarker requirements [4, 5].

Over a decade after their introduction, molecular tumor boards (MTBs) have emerged as an interdisciplinary approach to precision oncology, guiding individualized treatments based on molecular profiling [6]. Extensive diagnostic panels or whole‐exome sequencing require advanced bioinformatic and clinical resources to identify clinically actionable mutations, demanding interdisciplinary collaboration [7, 8]. Platforms such as the MTB Portal and cBioPortal aid in the interpretation of complex results [9, 10, 11, 12, 13]. Moreover, recent prospective initiatives have demonstrated the feasibility of highly integrative, multi‐omics tumor profiling to inform MTB decision‐making in melanoma [14]. However, such approaches rely on extensive infrastructure. They are not yet representative of routine clinical MTB workflows, and granular real‐world data on patient selection, timing, implementation, and the clinical impact of MTB recommendations in standard care settings remain scarce. This study retrospectively analyzes melanoma cases presented in institutional MTBs. By evaluating patient characteristics, molecular findings, treatment recommendations, implementation rates, and individual outcome measures, it aims to assess the role of MTBs in melanoma management and to identify barriers to optimizing their clinical impact.

## 2 Patients, Materials, and Methods

### 2.1 Patients

This study retrospectively analyzed 80 patients with advanced melanoma at the Department of Dermatology and Venereology of the University Medical Center Hamburg‐Eppendorf (43 patients) and the University Medical Center Freiburg (37 patients), who were presented to the institutional MTB between 2020 and 2024 at the respective centers and for whom follow‐up data were available. The institutional MTB is part of the two Centers for Personalized Medicine—Oncology (ZPM‐Oncology), either within the University Cancer Center Hamburg (UCCH) or within the Comprehensive Cancer Center Freiburg (CCCF), both Oncology Centers of Excellence recognized and funded by the German Cancer Aid (DKH). Data export included age groups, sex, and previously known tumor mutations. Further clinical data included the tumor stage at MTB referral, prior treatment lines, and melanoma subtype. Referral reasons to MTBs were extracted from free‐text commentaries. Molecular tumor data were derived from molecular analysis of tumor tissue at the time of MTB. This included whole‐exome sequencing (5 patients), Tru‐Sight Oncology 500 panel (Illumina, Berlin, Germany, 63 patients), and other smaller targeted panels. Data had undergone preprocessing, variant calling, annotation, and generation of Mutation Annotation Format (MAF) data before export. Tumor mutational burden (TMB), tumor purity, and ploidy were derived from the bioinformatic analysis of targeted next‐generation sequencing data as part of the institutional sequencing pipeline and imported together with the sample annotation file for downstream analysis. Somatic variant data were reported on the Tier classification system [15].

### 2.2 Statistical Analysis

To characterize molecular findings and investigate clinical associations, different bioinformatic platforms were used: For cohort characteristics, GraphPad Prism version 10.4.0 was used. Python in Google Colab was used for the analysis of time to MTB presentation and granular TMB analysis. MTB‐derived MAF data were analyzed with R in R Studio version 4.4.2, including the maftools package [16]. TCGA data analysis was conducted using MAF files from Broad Firehose (doi:10.7908/C11G0KM9) and the TCGA MC3 project [17]. Kaplan–Meier analysis was performed in Python using the lifelines library, and the log‐rank test was used to evaluate for statistical significance at *p* < 0.05. Patients with missing data for a given variable were excluded from the respective analysis. Patients who were lost to follow‐up were censored at the date of last known contact for time‐to‐event analyses. All analyses were considered exploratory. For visualization in Figure S1, ploidy values were categorized into diploid (< 2.5), triploid (2.5–3.5), tetraploid (3.5–4.0), and highly aneuploid (> 4.0). The progression‐free survival (PFS) ratio was calculated as the ratio between progression‐free survival on MTB‐guided therapy (PFS2) and progression‐free survival on the immediately preceding therapy line (PFS1), calculated on a per‐patient basis. A PFS ratio ≥ 1.3 was considered indicative of clinically meaningful benefit, as previously proposed by Von Hoff et al. [18].

## 3 Results

### 3.1 Patient Characteristics

**FIGURE 1:**
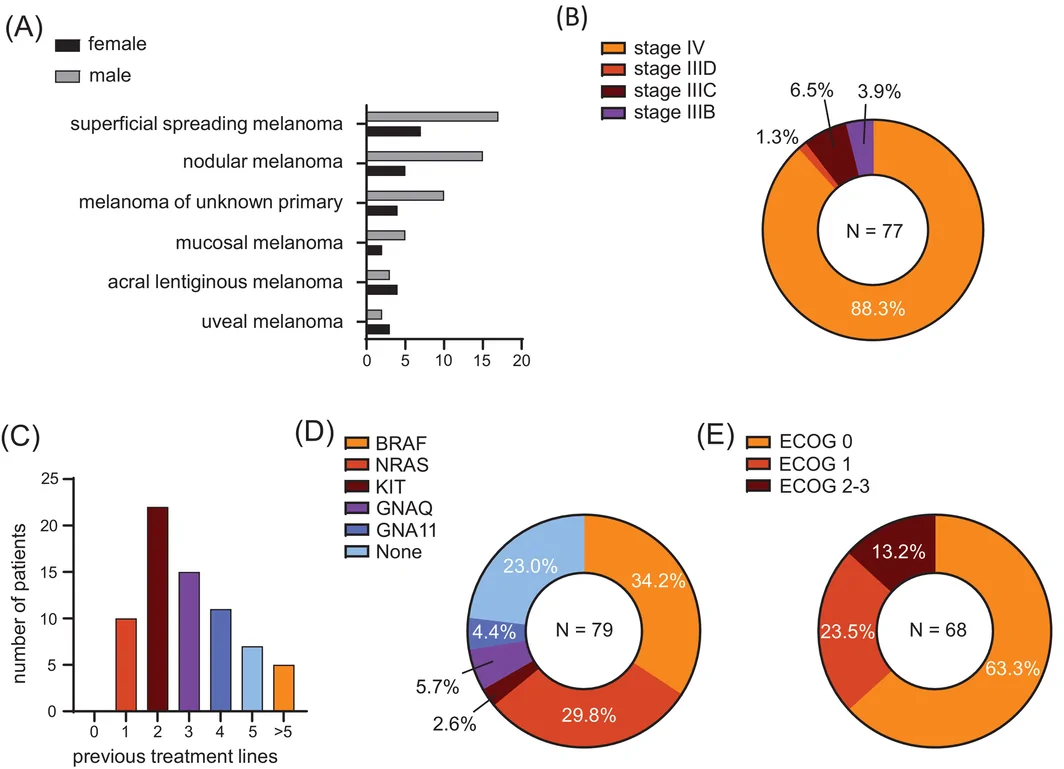
Clinical characteristics of patients presented to MTB. (A) Bar plot of melanoma subtype stratified by sex. (B) Percentage of patients presented at different AJCC‐v8 disease stages. (C) Number of previous treatment lines per patient upon MTB presentation. (D) Percentage of previously identified mutations upon MTB presentation and (E) Eastern Cooperative Oncology Group (ECOG) performance status.

A total of 80 patients with advanced melanoma were identified. The cohort displayed a diverse distribution of melanoma subtypes, with superficial spreading melanoma and nodular melanoma being the most frequent. Less common subtypes, including mucosal, acral lentiginous, and uveal melanoma, were observed at lower frequencies, with no significant sex‐based differences (Figure 1A). At the time of MTB presentation, the majority of patients (88.3%) had stage IV disease, while smaller proportions were classified as stage IIID (6.5%), stage IIIC (3.9%), and stage IIIB (1.3%) (Figure 1B). Most patients had undergone multiple previous treatment lines, with a median of three prior systemic therapies (range: 1–10) before referral (Figure 1C). All patients had previously received immune checkpoint inhibitor–based therapy prior to MTB presentation, most commonly combination therapy with ipilimumab and nivolumab or PD‐1 inhibitor–based regimens. In molecular routine testing before MTB, *BRAF* (34.2%), and *NRAS* (29.8%) were the most frequently detected mutations, followed by *KIT* (5.7%), *GNAQ* (4.4%), and *GNA11* (2.6%), while 23.0% of patients did not exhibit a detected driver mutation (Figure 1D). ECOG performance status 0 was observed in 63.3% of patients, with a median age of 53 years. Higher scores were observed in 23.5% (ECOG 1) and 13.2% (ECOG 2–3), respectively with a median age of 57 and 55 years (Figure 1E).

### 3.2 Timing and Reasons for MTB Referral

**FIGURE 2:**
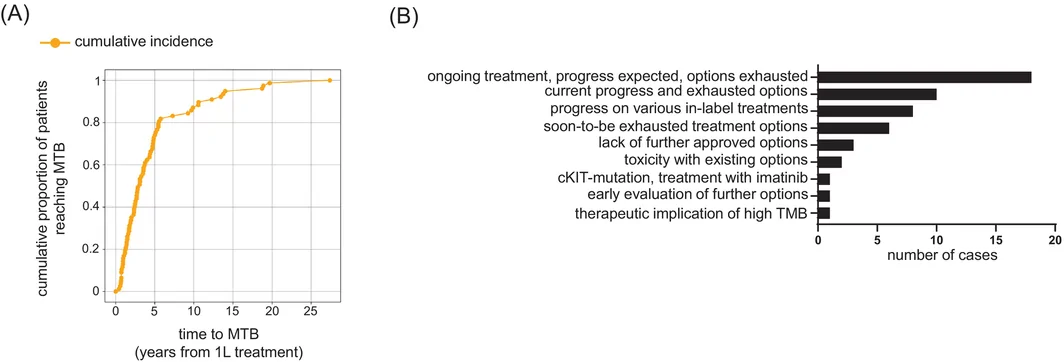
Time to MTB and reasons for presentation. (A) Cumulative incidence plot. Relative patient fraction presented to MTB, depending on time in years from first‐line treatment start. (B) Bar plot depicting reasons for presentation to MTB.

To evaluate the time to MTB presentation from the initiation of the first‐line melanoma treatment, a cumulative incidence plot was generated (Figure 2A). The median time to MTB presentation was 3.06 years from the initiation of first‐line melanoma treatment. Next, we investigated the reasons for the MTB presentation. The most common indications included ongoing treatment with expected progression and exhausted options, followed by current progression with no remaining standard treatment choices (Figure 2B). Other frequently observed reasons included progression on various in‐label treatments and soon‐to‐be exhausted treatment options. Less common indications included a lack of further approved options, treatment‐limiting toxicity, and specific molecular findings such as cKIT mutations, for which targeted approaches (e.g., imatinib) were considered. A small subset of patients was referred for early evaluation of potential future treatment avenues.

### 3.3 TMB Analysis

**FIGURE 3:**
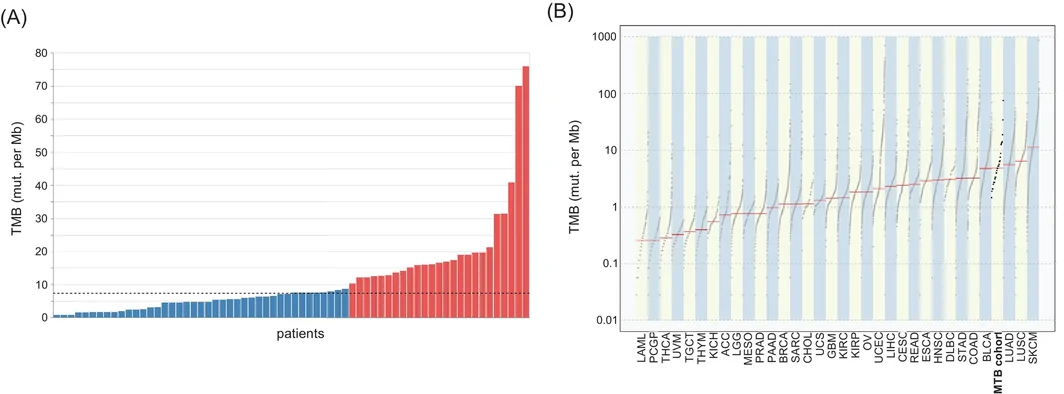
Tumor mutational burden analysis. (A) Bar plot of tumor mutational burden (TMB) in mutations per megabase for each patient. Red‐colored bars indicate TMB > 10. The horizontal dotted line indicates the median TMB of the cohort. (B) Logarithmic scatter plot of TCGA background data from Broad Firehose and TCGA MC3 project plotted alongside the data of this cohort (MTB cohort). TMB in mutations per megabase. LAML: acute myeloid leukemia, PCGP: pediatric cancer genome project, THCA: thyroid carcinoma, UVM: uveal melanoma, TGCT: testicular germ cell tumors, THYM: thymoma, KICH: kidney chromophobe, LG.G.: brain lower grade glioma, ACC: adrenocortical carcinoma, MESO: mesothelioma, PRAD: prostate adenocarcinoma, PAAD: pancreatic adenocarcinoma, BRCA: breast invasive carcinoma, SARC: sarcoma, CHOL: cholangiocarcinoma, UCS: uterine carcinosarcoma, GBM: glioblastoma multiforme, KIRC: kidney renal clear cell carcinoma, KIRP: kidney renal papillary cell carcinoma, LIHC: liver hepatocellular carcinoma, OV: ovarian serous cystadenocarcinoma, UCEC: uterine corpus endometrial carcinoma, READ: rectum adenocarcinoma, ESCA: esophageal carcinoma, HNCS: head and neck squamous cell carcinoma, DLBC: diffuse large B‐cell lymphoma, STAD: stomach adenocarcinoma, COAD: colon adenocarcinoma, BLCA: bladder urothelial carcinoma, MTB cohort: molecular tumor board cohort, LUAD: lung adenocarcinoma, LUSC: lung squamous cell carcinoma, SKCM: skin cutaneous melanoma.

To assess the TMB within the cohort, individual patient TMB values were analyzed, revealing a median TMB of 7.3 mutations per megabase (mut/Mb). A TMB above 10 Mut/Mb was identified in 37.88% of the cohort, which is based on the formal cut‐off used for TMB as biomarker according to panel‐NGS as approved in the KEYNOTE‐158 trial of Pembrolizumab of metastatic solid tumors (Figure 3A) [19]. To contextualize the TMB values observed in this cohort, a logarithmic scatter plot was generated, incorporating data from multiple cancer entities based on the TCGA MC3 dataset (Figure 3B). Cutaneous melanoma (SKCM) had the highest TMB among the included tumor types, consistent with its known genomic instability [20]. The MTB cohort exhibited lower TMB values compared to SKCM, positioning it closer to the BLCA (bladder cancer) group in the dataset. To improve comparability with the TCGA cutaneous melanoma (SKCM) dataset, uveal, and acral lentiginous melanoma cases were excluded from this comparison, as these subtypes are known to exhibit substantially lower TMB values.

### 3.4 Molecular Alterations and Complex Tumor Genomic Landscape Analysis

**FIGURE 4:**
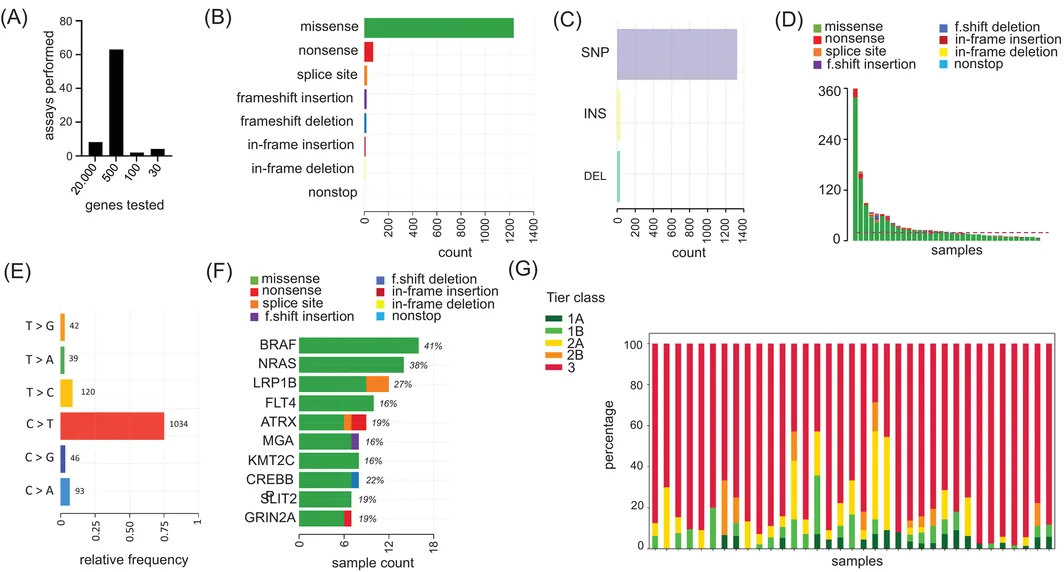
Molecular results of MTB analysis. (A) Approximate number of genes tested per assay performed for the MTB cohort. (B) Number of detected types of mutations in the MTB cohort. (C) The number of different variant types detected. SNP: Single nucleotide polymorphism, INS: Insertion; DEL: Deletion. (D) Number of Variants per patient detected. Color‐coding of different types of mutations. f.shift: Frameshift. Horizontal dotted line indicating median number of variants in MTB cohort. (E) Types of single nucleotide variant classes, showing the frequency of specific base substitutions in the MTB cohort. (F) Top 10 mutated genes in the MTB cohort with indicated frequency and color‐coding of the type of mutation. f.shift: Frameshift. (G) Percentage of identified mutations classified into Tier classes based on AMP/ASCO/CAP guidelines for each patient, for whom sufficient data were available.

Further characterizing the molecular landscape of MTB cases, molecular profiling assays were analyzed, revealing that most MTB cases were evaluated by comprehensive gene panels, such as whole‐exome sequencing or 500‐gene panels (Figure 4A). The majority of detected mutations were missense mutations (Figure 4B). Single nucleotide polymorphisms (SNPs) were the most frequent variant type (Figure 4C). The median number of variants per sample was 19 (0–359) (Figure 4D). Among single‐nucleotide variants (SNVs), C>T transitions were the most common, followed by T>C and other transition events (Figure 4E).

The most frequently mutated genes included *BRAF* (41%), *NRAS* (38%), and *LRP1B* (27%), with additional recurrent alterations in *FLT4*, *ATRX*, and *MGA* (Figure 4F). Finally, tier‐classified mutations were analyzed. Most individual variants were categorized as Tier 2 or Tier 3 alterations. However, at the patient level, Tier 1A or Tier 1B variants were detected in the majority of cases (Figure 4G).

To further explore the genomic characteristics of the cohort, an oncoplot was generated to illustrate the distribution of mutations alongside TMB classification and ploidy status (Figure S1). In addition to frequently mutated genes, a subset of tumors exhibited multi‐hit alterations, particularly in *BRAF, LRP1B, ATRX, FLT4*, and *KMT2C*. Most tumors in the cohort were highly aneuploid, with only a minority classified as diploid, triploid, or tetraploid, indicating widespread chromosomal alterations across the cohort (Figure S1).

### 3.5 Implementation of MTB Recommendations

**FIGURE 5:**
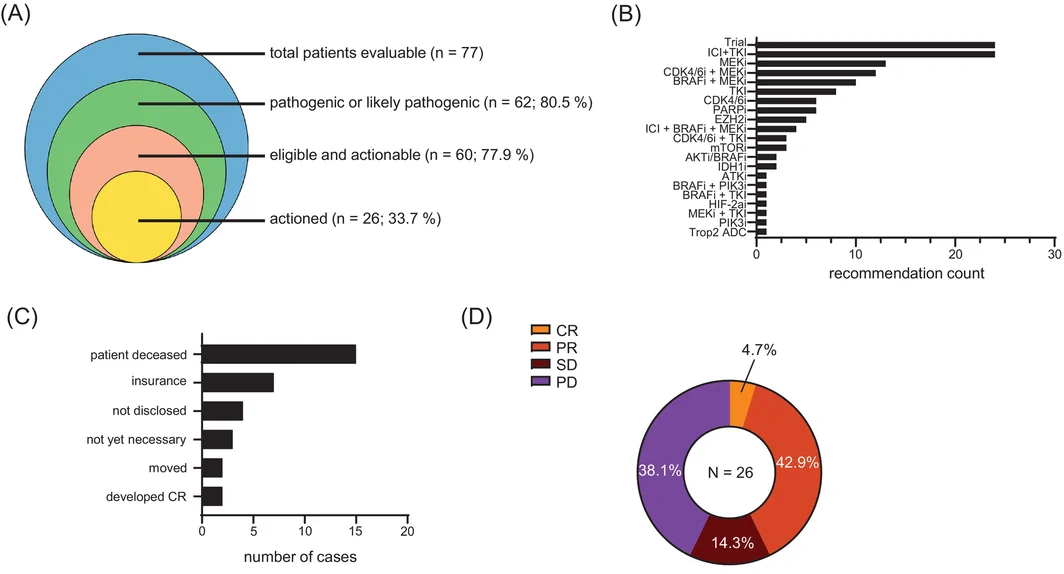
Implementation of MTB recommendations. (A) Venn diagram showing the number of patients evaluable for implementation analysis; the fraction of patients for which pathogenic or likely pathogenic variants were identified; out of these, cases with actionable variants and patients eligible to treatment targeting these; the fraction of patients for whom recommendations were finally implemented. (B) Treatments recommended in MTB. ICI: immune checkpoint inhibitor, TKI: tyrosine kinase inhibitor, MEKi: mek inhibitor, CDK4/6i: cyclin‐dependent kinase 4/6 inhibitor, BRAFi: Braf inhibitor, PARPi: Parp inhibitor, mTORi: mammalian target of rapamycin inhibitor, AKTi: akt inhibitor, HIF‐2ai: hypoxia‐induced factor 2 alpha inhibitor, PIK3i: proteinkinase 3 inhibitor, Trop2i: trophoblast cell‐surface antigen 2 inhibitor. (C) Bar plot depicting the reasons MTB‐recommended treatments were not implemented. (D) Percentage of patients exhibiting complete response (CR), partial response (PR), stable disease (SD), or progressive disease (PD) based on initial routine treatment response evaluation.

To investigate the implementation of MTB recommendations, a Venn diagram revealed that pathogenic or likely pathogenic alterations were identified in 80.5%, and 77.9% were eligible for targeted treatment. However, the recommended treatment was implemented in 33.7% only (Figure 5A). Clinical trials and ICI in combination with tyrosine kinase inhibitors were among the most frequently recommended treatments by MTB (Figure 5B). Implementation was often not realized due to early patient death or regulatory barriers (Figure 5C). When evaluating the initial response to the implemented treatment, that is, the first documented response after treatment initiation, data indicated that disease control was achieved in 69.2% of cases (Figure 5D).

### 3.6 Treatment Outcomes Following MTB Implementation

**FIGURE 6:**
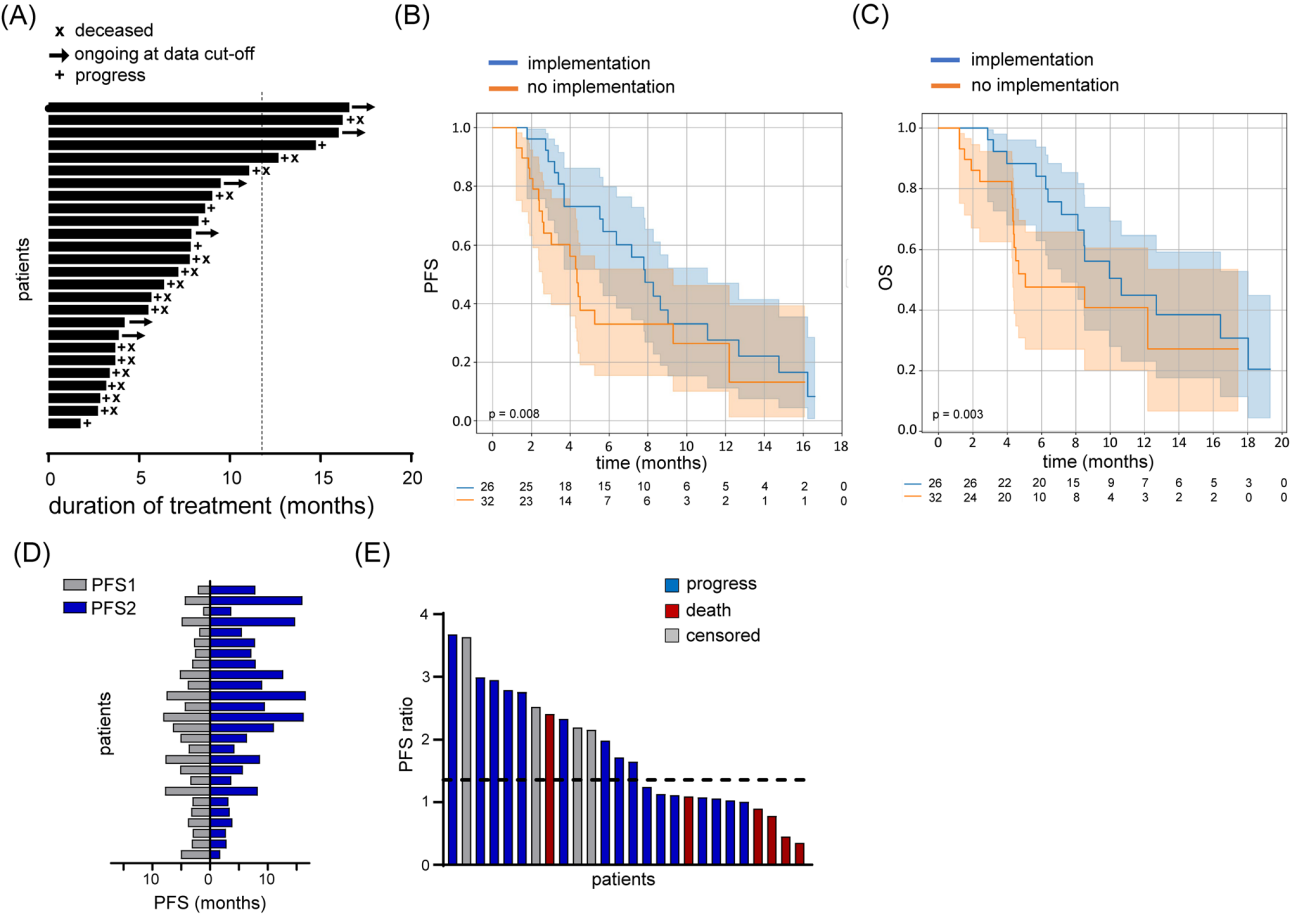
Individual treatment responses and Kaplan–Meier analysis. (A) Graphical illustration of individual cases with implemented MTB‐recommended treatments. Duration of treatment in months. The vertical dotted line indicates a treatment duration of over 12 months. X: Progress of the disease; arrow: Treatment ongoing at data cut‐off; +: Patient succumbed to death. (B, C) Kaplan–Meier analysis of (B) progression‐free survival (PFS) and (C) overall survival (OS) of patients with implemented MTB‐recommended treatment compared to evaluable patients without implementation of treatment. Two patients with actionable alterations but without implemented MTB therapy were excluded from the survival analysis due to missing follow‐up data, resulting in 32 evaluable patients in the non‐implemented group. Shaded areas represent 95% confidence intervals. Log‐rank test, significance level *p* < 0.05. (D) Mirrored bar plot showing PFS on the therapy preceding MTB discussion (PFS1, gray) and on MTB‐guided therapy (PFS2, blue) for individual patients. Patients are ordered according to decreasing PFS ratio. (E) Individual PFS ratios. Each bar represents one patient and is ordered by decreasing PFS ratio. The dashed horizontal line indicates a PFS ratio of 1.3.

The treatment trajectories of patients who received MTB‐recommended therapies were analyzed to assess disease progression, treatment duration, and survival outcomes (Figure 6A). While most patients eventually experienced disease progression, treatment implementation was associated with a notable delay in progression compared to those without new therapy. A subset of four patients remained on therapy for over 12 months, indicating the potential to achieve durable responses in some cases. Kaplan–Meier analysis was performed to assess the impact of implemented treatments versus evaluable cases in which no treatment was implemented (Figure 6B,C). Indeed, median PFS was more prolonged in cases where treatment was implemented (7.85 vs. 4.34 months, *p* = 0.008) (Figure 6B). Similarly, patients with implemented treatment exhibited longer overall survival (OS) compared to non‐implemented cases (10.64 vs. 5.06 months, *p* = 0.003) (Figure 6C). Baseline characteristics of patients with implemented versus non‐implemented MTB recommendations were compared (Table S1). Age, ECOG performance status, sex, melanoma subtype, AJCC stage, and number of prior treatment lines were similar between the two groups, and no statistically significant differences were observed (Table S1). Among patients who received MTB‐guided therapy, the median PFS ratio was 1.68, and 14 (53.8%) achieved a PFS ratio ≥ 1.3 (Figure 6D,E, Table S2).

## 4 Discussion

This study investigated the role of MTB in guiding treatment decisions for patients with advanced melanoma. Unlike other pan‐cancer reports, this exploratory bicenter study focuses specifically on patients with advanced melanoma treated at two tertiary referral centers in Germany. Most referrals were for stage IV melanoma (88.3%), with a median three‐year delay after first‐line treatment initiation. A delayed referral seems to have limited the feasibility of implementing MTB recommendations, aligning with prior findings that later genomic testing correlates with worse outcomes [21, 22, 23].

A pan‐cancer cohort identified 36.5% eligibility for actionable treatment, with only 8.9% of patients receiving therapy [24]. Our melanoma‐focused study shows higher eligibility (77.9%) and actioned cases (33.7%), likely due to broader panel testing and melanoma's high frequency of druggable targets. A Japanese cohort found 6% implementation, potentially due to smaller panel sizes [25]. Notably, the majority of our cohort exhibited Tier 1 variants of strong clinical significance, reinforcing their potential for actionable treatment [15, 26].

TMB analysis revealed a median of 7.3 mut/Mb, with 37.88% of cases above the TMB‐high threshold (10 mut/Mb), qualifying for Pembrolizumab monotherapy [27]. The MTB cohort exhibited lower TMB than cutaneous melanoma (SKCM) in TCGA MC3 but resembled bladder cancer (BLCA). Of note, uveal, and acral lentiginous melanoma cases were excluded from this comparison, as these subtypes are known to exhibit substantially lower TMB values. However, comparisons with TCGA datasets should be interpreted cautiously, as TCGA analyses are primarily based on whole‐exome sequencing whereas the present study relies on targeted panel sequencing, which may influence absolute TMB estimates. Given the high fraction of recommendations for actionable treatments, TMB did not directly account for mutation actionability. As expected, C‐to‐T transitions, which are typical of UV‐related damage such as in melanoma but not indicative of oncogenic NRAS or *BRAF* mutations, were the most prevalent mutations detected [28].

Patients were referred primarily due to exhausted treatment options or anticipated progression. While recommendations were formulated in the majority of cases, only 33.7% were implemented, often due to early patient death before treatment initiation. Late referral was the primary obstacle, as seen in other studies where deteriorating patient status was the main barrier to MTB adherence [23, 29, 30]. Our findings suggest that financial reimbursement issues may also have hindered implementation, consistent with a virtual expert survey [31] Global MTB turnaround times may also delay effective treatment [32, 33]. These findings highlight the potential importance of earlier MTB referral in order to increase the likelihood that targeted therapies based on MTB diagnostics and recommendations can be implemented. Among patients who received treatment based on MTB recommendations, a remarkable 69.2% achieved disease control, which is higher than in previous reports [26]. Clinical trial participation represented a frequent MTB recommendation in our cohort, highlighting the role of MTBs in identifying patients who may benefit from investigational therapies. However, the limited proportion of implemented trial recommendations also suggests that earlier referral to MTB may increase the likelihood of successful trial enrollment. Although most eventually progressed, Kaplan–Meier analysis revealed significantly longer PFS and OS than in patients in whom tumor board recommendations were not implemented. The observed survival benefit in patients with implemented MTB recommendations should be interpreted with caution, as non‐implementation was frequently related to rapid clinical deterioration or early death. However, baseline characteristics between groups were comparable, suggesting that the survival difference cannot be fully explained by measured baseline variables.

In our cohort, the median PFS ratio was 1.68%, and 53.8% of patients achieved a PFS ratio ≥ 1.3, suggesting clinically relevant benefit in a substantial proportion of patients receiving MTB‐guided therapy. This proportion appears comparatively high compared with other precision oncology studies, in which approximately 30%–40% of patients typically reach this threshold [18]. Previous studies have also shown that the degree of molecular matching between genomic alterations and administered therapies may influence clinical outcomes. For example, the I‐PREDICT study demonstrated that a higher matching score between molecular alterations and targeted therapies was associated with improved clinical benefit and higher rates of favorable PFS ratios [34]. However, several factors may influence the interpretation of our findings. In some cases, the preceding therapy line (PFS1) consisted of late‐line treatments such as dacarbazine (DTIC), which are known to be associated with relatively short PFS. This likely reflects the frequently late referral to MTB observed in our cohort and may partially contribute to higher PFS ratios when subsequent MTB‐guided therapies are administered. A multiomic approach could represent a promising avenue for better stratifying patients for individualized treatment strategies, potentially further improving outcomes [14]. However, a possible bias for reduced PFS and OS in non‐implemented cases could be the reason for non‐implementation: the death of patients before implementation was possible.

This study has further limitations: First, its exploratory, retrospective nature may introduce biases, though complete evaluable internal institutional cohorts minimize selection bias. Second, the study is limited by its bicenter design within two German tertiary referral centers.

Referral pathways, access to molecular diagnostics, clinical trial availability, reimbursement structures, and MTB workflows may differ substantially between institutions and healthcare systems. Therefore, the implementation rates and referral timing observed in this cohort may partially reflect center‐specific practices and should be interpreted with caution when extrapolating these findings to other countries or healthcare settings.

Another limitation of our study is that molecular profiling was primarily based on targeted next‐generation sequencing. While this approach enabled the detection of somatic mutations as well as selected structural alterations such as gene amplifications and gene fusions, broader genomic and transcriptomic alterations may not have been systematically captured. More comprehensive molecular profiling approaches may therefore further expand the spectrum of actionable alterations in MTB settings.

This bicenter real‐world study provides a melanoma‐specific, patient‐level evaluation of MTBs by systematically linking molecular findings, treatment recommendations, real‐world implementation, and clinical outcomes. It shows that although actionable recommendations are frequently generated, their implementation is often limited by late referral and regulatory barriers. Importantly, implementation of MTB‐guided therapies is associated with meaningful improvements in progression‐free and overall survival, underscoring the importance of timing as a key determinant of clinical impact. Our findings further highlight the importance of timely MTB referral. From a clinical perspective, meaningful benefit from MTB‐guided therapies should ideally translate into durable disease control and measurable improvements in progression‐free or overall survival. Achieving such benefit likely depends not only on the identification of actionable molecular alterations but also on timely referral and the patient's clinical condition at the time of MTB discussion. In several cases within our cohort, MTB recommendations could not be implemented due to advanced disease stage or clinical deterioration at the time of presentation. While our retrospective data do not allow the definition of a strict stage‐based threshold for MTB referral, earlier molecular profiling in selected high‐risk patients, timely referral to MTB prior to loss of treatment eligibility, and closer integration of MTB discussions with clinical trial screening may increase the likelihood of successful treatment implementation. Furthermore, predefined institutional referral pathways may facilitate earlier MTB presentation in suitable patients, and improved interdisciplinary coordination between oncology, dermatology, pathology, and molecular diagnostics may help reduce delays between molecular testing, MTB recommendation, and treatment initiation. In addition, improving the feasibility and adherence to MTB recommendations may represent an important factor for maximizing the clinical impact of precision oncology strategies.

## Author Contributions

**Glenn Geidel:** conceptualization, methodology, software, data curation, investigation, formal analysis, validation, visualization, project administration, resources, writing – original draft, writing – review and editing, supervision. **Theresa Lowinus:** investigation, data curation, formal analysis, writing – review and editing. **Patrick Metzger:** conceptualization, methodology, project administration, resources, writing – review and editing, supervision. **Rebecca Czurda:** data curation, writing – review and editing. **Vincent Schipperges:** data curation, formal analysis, writing – review and editing. **Ulrike Paul:** resources, writing – review and editing. **Alessandra Rünger:** resources, writing – review and editing. **Julian Kött:** resources, writing – review and editing. **Isabel Heidrich:** resources, writing – review and editing. **Saskia Lehr:** resources, writing – review and editing. **Julia Kühn:** resources, writing – review and editing. **Heiko Becker:** resources, writing – review and editing. **Cornelius Miething:** resources, writing – review and editing. **Martin Werner:** resources, writing – review and editing. **Silke Laßmann:** resources, writing – review and editing. **Justus Duyster:** resources, writing – review and editing. **Tilman Brummer:** resources, writing – review and editing. **Ronald Simon:** resources, writing – review and editing. **Winfried Alsdorf:** resources, writing – review and editing. **Maximilian Christopeit:** resources, writing – review and editing. **Carsten Bokemeyer:** resources, writing – review and editing. **Kilian Eyerich:** resources, writing – review and editing. **Stefan W. Schneider:** resources, writing – review and editing. **David Rafei:** resources, writing – review and editing. **Melanie Börries:** resources, writing – review and editing. **Frank Meiss:** conceptualization, writing – review and editing, resources, methodology, project administration, supervision. **Christoffer Gebhardt:** conceptualization, methodology, project administration, resources, writing – review and editing, supervision.

## Ethics Statement

The data reported herein has been approved by the Ethics Committee of the Hamburger Ärztekammer (2025‐300584‐WF).

## Conflicts of Interest

G.G. declares consulting fees from advisory or data safety boards, honoraria for lectures and educational events, and travel support by Almirall Hermal, BMS, Delcath Systems, Immunocore, Regeneron Pharmaceuticals, MSD, SUN‐Pharma, Pierre Fabre, and Janssen‐Cilag, outside the submitted work. G.G. has received grants from Regeneron Pharmaceuticals and Delcath Systems outside the submitted work. G.G. is a board member of SCOPE, unpaid. J.K. has received honoraria from Bristol‐Myers Squibb, UCB, Sun Pharma, Sanofi, Novartis, Beiersdorf AG, doctorderma Online Hautarzt and Melatech ApS, outside the submitted work; J.K. has received travel support from SUN Pharma and Pierre Fabre, outside the submitted work. T.B. received speaker honoraria from Pierre Fabre and the European Society for Medical Oncology (ESMO). W.A. has received honoraria from Astellas, AstraZeneca, GSK, and Johnson&Johnson, served as a consultant for Johnson&Johnson, received travel expenses from BioNTech, Immatics, and Johnson&Johnson and obtained research funding for clinical trials by Affimed and BioNTech (received by the institution); all outside of the submitted work. M.C. received honoraria, travel, and research support from BeOne/BeiGene, JAZZ, GSK, Roche, Tillotts, AstraZeneca, AbbVie, Janssen Cilag, Ciba Geigy, Boehringer Ingelheim, Pfizer, MSD, Takeda, Medac, Servier, and BMS. He is the co‐holder of US‐Patent (PCT/US2014/72474) “EPIGENETIC STEM CELL COMMITMENT ASSOCIATED SIGNATURE.” C.B. declares consulting fees from advisory or data safety boards, honoraria for lectures and educational events, and travel support by AOK Germany, Astra Zeneca, Bayer Healthcare, BioNTech, Lindis Biotech, Sanofi Aventis GSO CRO, med update, Merck Serono, Roche Pharma, Dayichi‐Sankyo, all outside the submitted work. C.B. declares participation on advisory boards and leadership roles for the German Society of Hematology and Oncology (DGHO), the Hamburg Cancer Society (HKG), the German Cancer Society (DKG), the National Network of German Cancer Centers (CCC)/German Cancer Aid (DKH), and the Northern German Society of Internal Medicine (NWGIM), all outside the submitted work. F.M. served as consultant and/or has received honoraria from Novartis, Bristol‐Myers Squibb, Merck Sharp & Dohme, Pierre Fabre, Sanofi Genzyme, Sun Pharma, and travel support from Novartis, Sun Pharma, Pierre Fabre and Merck Sharp & Dohme, outside the submitted work. C.G. is a board member of the DeCOG (ADO), unpaid; C.G. is a board member of the Hiege Stiftung, unpaid. C.G. is board member of the Roggenbuck‐Stiftung, unpaid. C.G. is board member of the EADO, unpaid; C.G. is board member of the MWS, unpaid. C.G. is founder of Dermagnostix and Dermagnostix R&D. C.G. has received honoraria by Almirall, Biofrontera, BMS, Delcath, Immunocore, Janssen, Medscape, MSD, NAOS, Novartis, Onkowissen, Pierre Fabre, Regeneron, Sanofi, SUN Pharma, Sysmex. C.G. has received travel expenses by BMS, Pierre‐Fabre, SUN Pharma. C.G. has been a member of the advisory board of Almirall, Beiersdorf, BioNTech, BMS, Delcath, ImCheck, Immunocore, Immatics, Iovance, Moderna, MSD, Novartis, Pierre Fabre, Regeneron, Sanofi, SkylineDX, SUN Pharma, Sysmex. The other authors declare no conflicts of interest.

## Supporting information


**Table S1:** Baseline characteristics of patients with implemented versus non‐implemented Molecular Tumor Board (MTB) treatment recommendations. Patient characteristics at the time of MTB presentation were compared between patients in whom MTB recommendations were implemented (*n* = 26) and those in whom recommendations were not implemented (*n* = 32). Data are presented as mean (range) for continuous variables and number (percentage) for categorical variables. *p* values were calculated using the Mann–Whitney *U* test for continuous variables and Fisher's exact test for categorical variables. ECOG, Eastern Cooperative Oncology Group performance status; AJCC, American Joint Committee on Cancer; ALM, acral lentiginous melanoma; MUP, melanoma of unknown primary; MMM, mucosal malignant melanoma; NMM, nodular malignant melanoma; SSM, superficial spreading melanoma; UM, uveal melanoma.
**Table S2:** Patient‐level overview of MTB‐guided therapies. Overview of clinical characteristics, selected molecular alterations, MTB treatment recommendations, implemented therapies, best response, and progression‐free survival (PFS) metrics for patients who received MTB‐guided therapy. PFS1 represents PFS on the therapy line immediately preceding MTB discussion, whereas PFS2 represents PFS on MTB‐guided therapy. The PFS ratio was calculated as PFS2 divided by PFS1. Event status indicates whether PFS2 ended with progression, death, or censoring at the last follow‐up. Depicted molecular alterations represent selected variants discussed during MTB evaluation. ALM, acral lentiginous melanoma; MUP, melanoma of unknown primary; MMM, mucosal malignant melanoma; NMM, nodular malignant melanoma; SSM, superficial spreading melanoma.
**Figure S1:** Oncoprint analysis. Oncoprint plot showing the mutational landscape of the MTB cohort. Each row represents a gene, with colored squares indicating different mutation types. The top bar plot displays the total number of mutations per sample, while the right bar plot shows the frequency of mutations per gene. Tumor mutational burden (TMB) status dichotomized and ploidy is indicated in the bottom annotation.

## Data Availability

The data that support the findings of this study are available upon reasonable request from the corresponding authors.
