# Supplementary material for: Molecular Tumor Boards in Malignant Melanoma: Uncovering Challenges and Opportunities in a Bicenter Retrospective Analysis in Germany
Source: Int J Cancer. 2026 May 15;159(9):2290–9. doi: 10.1002/ijc.70554 (PMC13548000; doi:10.1002/ijc.70554)
Supplement: Supplementary file 1 [file IJC-159-2290-s001.pdf]

Supplementary Material

Title: Molecular Tumor Boards in Malignant Melanoma: Uncovering Challenges and Opportunities in a Bicenter Retrospective Analysis in Germany

Authors: Glenn Geidel, Theresa Lowinus, Patrick Metzger, Rebecca Czurda, Vincent Schipperges, Ulrike Paul, Alessandra Rünger, Julian Kött, Isabel Heidrich, Saskia Lehr, Julia Kühn, Heiko Becker, Cornelius Miething, Martin Werner, Silke Laßmann, Justus Duyster, Tilman Brummer, Ronald Simon, Winfried Alsdorf, Maximilian Christopeit, Carsten Bokemeyer, Kilian Eyerich, Stefan W. Schneider, David Rafei, Melanie Börries, Frank Meiss, Christoffer Gebhardt

Table of contents:

(1) Supplementary Table S1 ……………………………………………………………………. page 2
(2) Supplementary Table S2 ……………………………………………………………………. page 3
(3) Supplementary Figure S1 …………………………………………………………………. page 5

(1) Supplementary Table S1

	Implemented (n = 26)	Not implemented (n = 32)	P value
**Age, mean (range)**	**54 (30-73)**	**56 (18-83)**	**0.62**
**ECOG status**			**0.41**
0	12 (46.2%)	19 (59.4)	
1	7 (26.9%)	5 (15.6%)	
2	1 (3.8%)	3 (9.3%)	
3	1 (3.8%)	2 (6.3%)	
Unknown	5 (19.2%)	3 (9.4)	
**Sex**			**0.28**
Male	19 (73.1%)	19 (59.4%)	
Female	7 (26.9%)	13 (40.6%)	
**Melanoma subtype**			**0.12**
ALM	4 (15.4%)	3 (9.4%)	
MUP	4 (15.4%)	4 (12.5%)	
MMM	3 (11.5%)	2 (6.3%)	
NMM	3 (11.5%)	12 (37.5)	
SSM	12 (46.2%)	9 (28.1)	
UM	0 (0%)	2 (6.3%)	
**Disease stage (AJCC v-8)**			**0.36**
IIIB	1 (3.8%)	4 (12.5%)	
IIIC	1 (3.8%)	2 (6.3%)	
IIID	0 (0%)	1 (3.1%)	
IV	24 (92.3%)	25 (78.1%)	
**Prior treatment lines**			**0.87**
0	0 (0%	0 (0%)	
1	6 (23.1%)	4 (12.5%)	
2	14 (53.8%)	20 (62.5%)	
3	3 (11.5%)	4 (12.5%)	
4	1 (3.8%)	2 (6.3%)	
5	1 (3.8%)	1 (3.1%)	
>5	1 (3.8%)	1 (3.1%)	

Table S1: Baseline characteristics of patients with implemented versus non-implemented Molecular Tumor Board (MTB) treatment recommendations. Patient characteristics at the time of MTB presentation were compared between patients in whom MTB recommendations were implemented (n = 26) and those in whom recommendations were not implemented (n = 32). Data are presented as mean (range) for continuous variables and number (percentage) for categorical variables. P values were calculated using the Mann–Whitney U test for continuous variables and Fisher's exact test for categorical variables. ECOG, Eastern Cooperative Oncology Group performance status; AJCC, American Joint Committee on Cancer; ALM, acral lentiginous melanoma; MUP, melanoma of unknown primary; MMM, mucosal malignant melanoma; NMM, nodular malignant melanoma; SSM, superficial spreading melanoma; UM, uveal melanoma.

(2) Supplementary Table S2

patient	melanoma subtype	previous therapy	selected molecular alterat	MTB treatment recommendation	implemented therapy	best response	PFS 1	PFS2	PFS ratio	PFS2 event status
P1	SSM	pembrolizumab	NRAS Q61R	pembrolizumab + lenvatinib; trial inclusion; ribociclib + binimetinib	pembrolizumab + lenvatinib	PD	5.06	1.77	0.35	death
P2	MMM	ipilimumab + nivolumab	FBXW7 T15_G16insP; SF3B1 R625H	everolimus + temozolomide	everolimus + temozolomide	PD	6.04	2.73	0.45	death
P3	NMM	encorafenib + binimetinib	BRAF V600E; BRAF T599I; NRAS Q61K; CDKN2A R58*	ribociclib + binimetinib	ribociclib + binimetinib	PD	3.19	2.86	0.90	death
P4	SSM	encorafenib + binimetinib	BRAF V600E; PIK3CA E542K; CCND3 amplification; CCNE1 amplification; CDK6 amplification	dabrafenib + trametinib + pembrolizumab; pembrolizumab + lenvatinib; AKTi + BRAFi or alpelisib + BRAFi; CDK4/6i	pembrolizumab + lenvatinib	PD	4.07	3.19	0.78	death
P5	NMM	DTIC	EZH2 Y646C; NRAS Q61K	pembrolizumab + lenvatinib; tazemetostat; ribociclib + binimetinib	pembrolizumab + lenvatinib	PD	3.29	3.38	1.03	progress
P7	MUP	trametinib	ERBB2 R756T	trial inclusion; MEKi	binimetinib	PD	3.42	3.68	1.08	progress
P6	MMM	ipilimumab + nivolumab	PIK3CA E545K	alpelisib	alpelisib	PD	2.23	3.68	1.65	progress
P8	NMM	paclitaxel	AGK–BRAF fusion	MEKi	trametinib	PD	3.84	3.88	1.01	progress
P9	SSM	ipilimumab + nivolumab	NRAS Q61K; ATM Y175fs	binimetinib (+/- ribociclib)	binimetinib	PR	3.71	4.20	1.13	progress
P10	SSM	encorafenib + binimetinib	BRAF V600E; PSMA1–MLLT3 fusion	binimetinib + encorafenib + pembrolizumab	binimetinib + encorafenib + pembrolizumab	PR	1.87	5.52	2.95	progress
P11	MUP	pembrolizumab + lenvatinib	BRAF V600E; RAC1 P29S; PTEN L140*	trial inclusion; mTORi; Akti; PARPi	trial inclusion	PD	5.19	5.68	1.09	death
P12	ALM	DTIC	KIT D820Y; CDK4 amplification; CBL Y371C; ATM variant; CHEK2 variant	trial inclusion; pembrolizumab + lenvatinib; olaparib; ripretinib; palbociclib	ripretinib	SD	5.12	6.37	1.24	progress
P13	MMM	nivolumab	BRAF D594G	sorafenib + trametinib	sorafenib + trametinib	SD	2.60	7.16	2.76	progress
P14	MUP	ipilimumab + nivolumab	CDK4 amplification; MDM2 amplification	trial inclusion; CDK4/6i	palbociclib	SD	2.79	7.79	2.79	progress
P15	ALM	DTIC	NRAS Q61R; GNA11 R183C	pembrolizumab + lenvatinib; trial inclusion; ribociclib + binimetinib	pembrolizumab + lenvatinib	SD	2.14	7.85	3.68	progress
P16	MUP	ipilimumab + nivolumab	NRAS Q61R; APC Q222*	trial inclusion; pembrolizumab + lenvatinib; ribociclib + binimetinib	pembrolizumab + lenvatinib	PR	3.12	7.88	2.53	censored
P17	ALM	DTIC	BCL2L11 W93; RAD50 frameshift; FGF/FGFR copy number alteration	trial inclusion; futibatinib; PARPi; CDK4/6i	futibatinib	SD	7.79	8.28	1.06	progress
P18	SSM	nivolumab	NRAS Q61R; ALK W615; RB1 Q597; GRIN2A W634; TGFBR2 E82K; CTNNB1 R661G; IKZF1 G169E; SUFU M177I	ribociclib + binimetinib; pembrolizumab + lenvatinib	ribociclib + binimetinib	PR	7.75	8.64	1.11	progress
P19	SSM	DTIC	NRAS Q61K	trial inclusion; ribociclib + binimetinib; belvarafenib + cobimetinib; pembrolizumab + lenvatinib	pembrolizumab + lenvatinib	SD	3.88	9.03	2.33	progress
P20	SSM	ipilimumab + nivolumab	BRAF G469A; BRAF L584F	trial inclusion; dabrafenib + trametinib; pembrolizumab + lenvatinib	pembrolizumab + lenvatinib	SD	4.40	9.49	2.16	censored
P21	SSM	pembrolizumab	BRAF V600E; PTEN G129R	BRAFi + MEKi; mTORi	encorafenib + binimetinib	PR	6.44	11.07	1.72	progress
P22	ALM	DTIC	BRAF V600E; GNAQ Q209P; PBRM1 R1185X	trial inclusion; tazemetostat; pembrolizumab + lenvatinib	trial inclusion	SD	5.26	12.68	2.41	death
P23	SSM	nivolumab	BRAF V600E	pembrolizumab + lenvatinib; dabrafenib + trametinib	dabrafenib + trametinib	CR	4.93	14.75	2.99	progress
P24	SSM	DTIC	CDK amplification; FMN1–BRAF fusion; ERBB3 amplification	trial inclusion	trial inclusion	PR	4.40	16.00	3.63	censored
P25	SSM	ipilimumab + nivolumab	NRAS Q71K; CTNNB1 variant	pembrolizumab + lenvatinib; ribociclib + binimetinib	pembrolizumab + lenvatinib	PR	8.18	16.23	1.98	progress
P26	SSM	pembrolizumab	MAP2K1 del105–106; FANCI M525V; NOTCH1 R912W; PTPRT Q1199*	MEKi	cobimetinib	PR	7.56	16.59	2.20	censored

Table S2: Patient-level overview of MTB-guided therapies. Overview of clinical characteristics, selected molecular alterations, MTB treatment recommendations, implemented therapies, best response, and progression-free survival metrics for patients who received MTB-guided therapy. PFS1 represents progression-free survival on the therapy line immediately preceding MTB discussion, whereas PFS2 represents progression-free survival on MTB-guided therapy. The PFS ratio was calculated as PFS2 divided by PFS1. Event status indicates whether PFS2 ended with progression, death, or censoring at the last follow-up. Depicted molecular alterations represent selected variants discussed during MTB evaluation. ALM, acral lentiginous melanoma; MUP, melanoma of unknown primary; MMM, mucosal malignant melanoma; NMM, nodular malignant melanoma; SSM, superficial spreading melanoma.

(3) Supplementary Figure S1

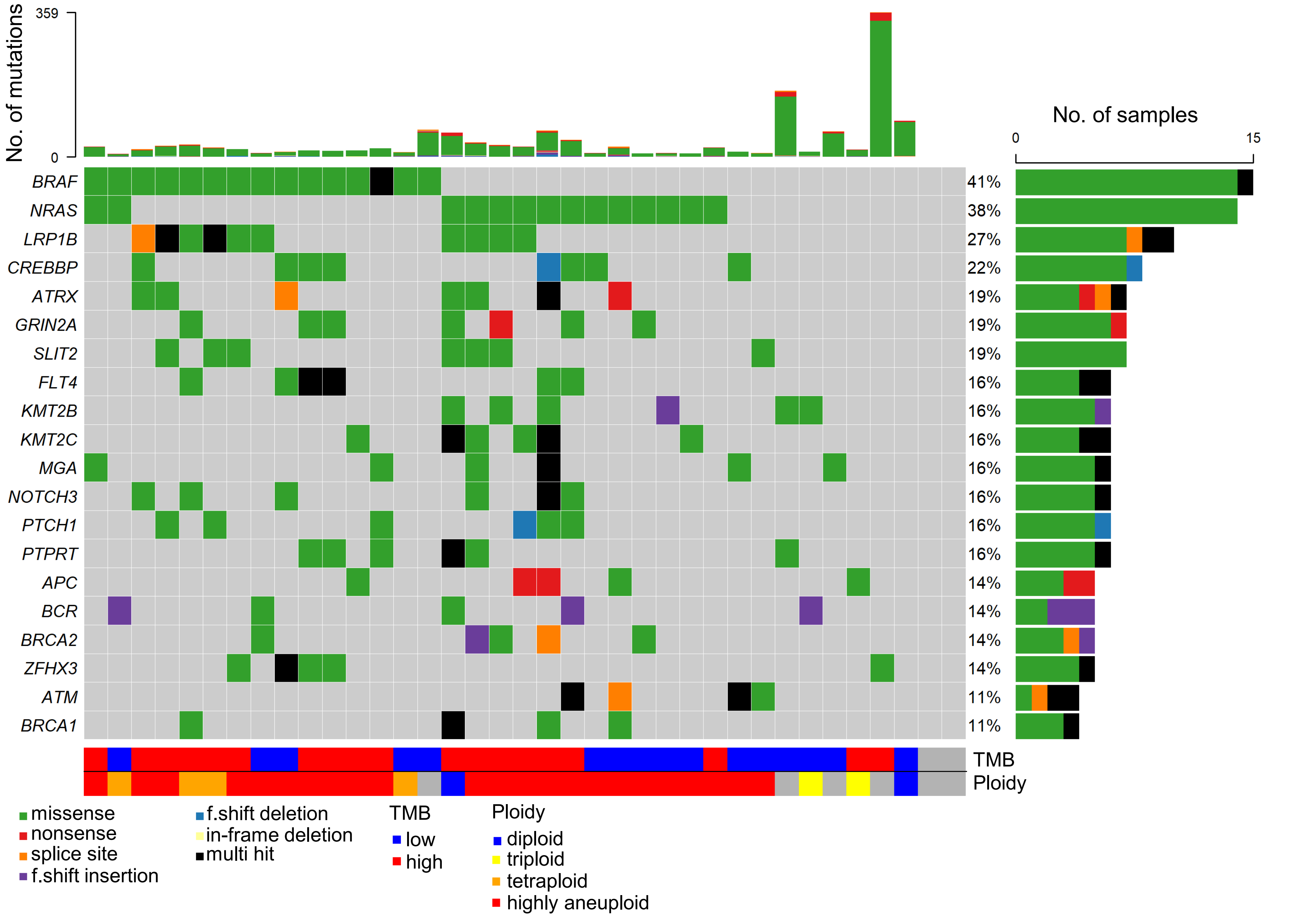

Figure S1: Oncoprint analysis. Oncoprint plot showing the mutational landscape of the MTB cohort. Each row represents a gene, with colored squares indicating different mutation types. The top bar plot displays the total number of mutations per sample, while the right bar plot shows the frequency of mutations per gene. Tumor mutational burden (TMB) status dichotomized and ploidy is indicated in the bottom annotation.
